# Cervical cancer screening patterns among HIV-positive women in Estonia: a population-based retrospective cohort study

**DOI:** 10.1186/s12885-021-08076-0

**Published:** 2021-04-01

**Authors:** Anna Tisler, Sven Erik Ojavee, Piret Veerus, Pilleriin Soodla, Anneli Uusküla

**Affiliations:** 1 Institute of Family Medicine and Public Health, University of Tartu, Tartu, Estonia; 2 Department of Computational Biology, University of Lausanne, Lausanne, Switzerland; 3 National Institute for Health Development, Tallinn, Estonia; 4 Department of Internal Medicine, Tartu University Hospital, Tartu, Estonia; 5 Institute of Clinical Medicine, University of Tartu, Tartu, Estonia

**Keywords:** Cervical cancer, Screening, HIV, Papanicolaou test, Human papillomavirus

## Abstract

**Background:**

The World Health Organisation (WHO) calls for the elimination of cervical cancer (CC) as a public health issue. To achieve elimination, efforts must be aligned and accelerated. Women living with HIV (WLWH) have excess risk for developing, and dying from, CC over the general population. Estimates of cervical cancer screening programme coverage in Eastern European countries that have experienced HIV epidemics since the early 2000’s are scarce.

**Method:**

This population-based retrospective study uses a healthcare administrative database and follows cohorts of all WLWH in a ratio of 1:3 randomly matched (age, region) HIV negative women from 2009 to 2018. Annual and longitudinal (over the whole study period) coverage for cervical cancer screening (opportunistic, organised, HIV specific) and adjusted odds ratios (AORs) for longitudinal screening coverage predictors were estimated from 2009 to 2018.

**Results:**

Among WLWH and HIV-negative women, the mean annual coverage with opportunistic screening was 61.45 and 65.59%; and organised screening was 20.4 and 28.7%, respectively (both: *p* < 0.00001). 19.01% (95% CI 18.05–19.97) HIV-negative and 13.9% (95% CI 12.35–15.45) WLWH were longitudinally covered with organised cervical cancer screening. Among WLWH, the mean annual HIV-specific cervical cancer screening coverage was 49.4, and 24.3% were longitudinally covered. Longitudinal coverage with HIV-specific cervical cancer screening was inversely associated with age, hepatitis C virus (HCV) co-infection (AOR 0.754, 95% CI 0.619, 0.916), not having insurance (AOR 0.331, 95% CI 0.264, 0.412), drug abuse (AOR 0.459, 95% CI 0.336, 0.618) and higher among those retained in HIV care (AOR 1.972, 95% CI 1.615, 2.410). Among HIV-negative women, longitudinal coverage with organised cervical cancer screening was inversely associated with residence in the region and higher among older women.

**Conclusions:**

Our results highlight unacceptably low coverage of cervical cancer screening of WLWH in Estonia. There is need for dedicated cervical cancer screening efforts for WLWH considering the high cancer risk and rate in the study population.

## Background

Ground breaking medical advances in recent decades have opened up the prospect of eliminating cervical cancer (CC). Until now, CC has been the fourth most common cancer in women [1] and the leading cause of cancer-related deaths among women in many countries. As such, this disease clearly illustrates the impact of inequality upon women’s health.

The acquisition and persistence of human papillomavirus (HPV) infection has increased among women living with HIV (WLWH), as well as the increased risk of invasive CC [2]. CC incidence among WLWH has been shown to be four times higher than that of HIV-negative women (incidence rate of 26 and 6 per 100,000 respectively) [3]. As a consequence, international guidelines recommend more frequent CC screening among WLWH [4].

Organised screening programmes have been remarkably successful in reducing the incidence and mortality of CC, while opportunistic testing varies in its effectiveness [5]. Failure to undergo recommended routine screening has been identified as the most significant risk for the development of CC, both in WLWH and in the general population (in countries where routine CC screening programmes are in place) [6].

Disparities in CC incidence and mortality in Europe between Central/Eastern and Scandinavian/Western European countries are well documented and long-standing. In Estonia, the CC incidence is over two times higher than in neighbouring Northern European/Scandinavian countries (Sweden 9.0, Finland 4.7) [7]. Furthermore, in Estonia, CC incidence is increasing (2012, 19.6 and 2018, 22.5 per 100,000), mortality is stagnating, and stage distribution at the time of diagnosis has shifted towards later stages. The failure of CC screening in Estonia has been attributed to low screening uptake (less than 50% in 2016) and the insufficient quality of the Pap test-based programme [8].

In 2018, of the 50,000 new HIV diagnoses among women of all ages in the WHO European Region, the majority (86%) were in the Eastern sub-region [9]. In the same year, Estonia had the third highest rate of newly diagnosed HIV cases in the European Union (EU) (Estonia 14.4/100,000 compared to an EU average of 5.1) [10].

Knowledge on cervical cancer screening coverage, and factors related to deficient screening, in European countries experiencing HIV epidemics is extremely limited.

The aim of this study was to assess the CC screening coverage among WLWH and in the general population in Estonia and to identify risk factors associated with low coverage in WLWH in order to guide HIV-specific CC screening strategies, if needed.

## Methods

### Overview

In this population-based retrospective cohort study, data on CC screening attendance and related factors of HIV-infected women were compared with age-matched, un-infected women.

### The cervical cancer screening programme in Estonia

An organised population-based CC screening programme (using conventional cytology, Papanicolaou test) has been in place in Estonia since 2006. Organised screening targets women in the age range 30–55 years, with a screening interval of 5 years across the whole period. Screening is free of charge, and screening invitations are only sent to women with health insurance. The screening invitation includes information of all clinics where screening services are offered. Specially trained midwives conduct the smear tests in clinics that participate in the programme [11].

### Data source

The population of Estonia is approximately 1,315,000 [12]. Universal public health insurance covers > 94% and has been stable since the inception of the insurance system in 2000. The vast majority of the population, including children and the elderly, are covered by the compulsory health insurance scheme. The 5% who are uninsured are mostly of working-age (20–60 years) who are economically inactive or unemployed [13]. The Estonian Health Insurance Fund (EHIF) is the core purchaser of healthcare services in Estonia, covering healthcare costs for insured people and also managing services for uninsured citizens. The EHIF has maintained a complete record of health care services. The EHIF electronic database contains personal information (gender, age at health care service utilisation), health care utilisation (date of service, primary and other diagnoses, treatment type (in- or outpatient), specialty of the provider), and the date of death. As EHIF reimburses health care providers on a fee-for-service basis, the database is considered to be relatively complete.

#### Identification of women living with HIV

Health care utilisation data for WLWH between 1st January 2009 and 30th June 2018 were included. The case definition of living with HIV was based on the HIV care specific diagnosis codes (International Classification of Diseases, Tenth Revision (ICD-10): B23.0; B23.1, Z21; F02.4, B20-B24) on any of the EHIF claims over the period of observation. The index date of diagnosis was defined as the first day of care indicated in the first claim with the HIV identifying diagnosis code.

#### Identification of general population women

The sample frame included all insured individuals, including those with no record of receiving healthcare services. WLWH were randomly matched by region and age (year of birth) in a 1:3 ratio to women in the general population. By definition, control group women were alive and had no evidence of HIV infection at the case patient’s (WLWH) index date (the date of the 1st health claim with and HIV indicative ICD-10 code).

For study purposes, study subjects were assigned a unique identifier decoupled from personal identification information to enable longitudinal tracking of care and mortality while maintaining patient privacy. Data on women younger than 16 years at the start of the study, and women with a history of CC, were excluded.

#### Defining screening episodes

To assess the study population coverage with CC, the following definitions were used.

First, we differentiated opportunistic and organised screenings. Opportunistic screening is spontaneous, depending on the initiative of the individual woman or her doctor. An opposite of this screening approach is organised screening, i.e. systematic, organised and population-based screening for early cancer or cancer precursor detection. In the data analysis, the two types of screening were distinguished by the codes from health care claim, and we present coverage estimates separately for general population, and WLWH.

An organised CC screening episode is defined on the bases of a health claim with the ICD-10 diagnosis code Z12.4 (Encounter for screening for malignant neoplasm of cervix). This diagnosis code (Z12.4) is specifically used by the organised screening services in Estonia.

An opportunistic screening episode is defined on the bases of a Pap test specific service code on a healthcare claim without the ICD-10 code Z12.4.

For both, WLWH and matched control the follow-up time started from the index date. We followed all study subjects (belonging to the WLWH and general population group) from the index date until the study end (30th June 2018), diagnosis date of CC, date of death and HIV diagnosis in control group.

We estimated annual and longitudinal coverage estimates for both screening types (separately for WLWH and women from the general reference population).

### Annual coverage

“For the purposes of this analysis, we define the annual coverage the percentage of women each year who have less than 5 years since their last Pap test.

**Fig. 1:**
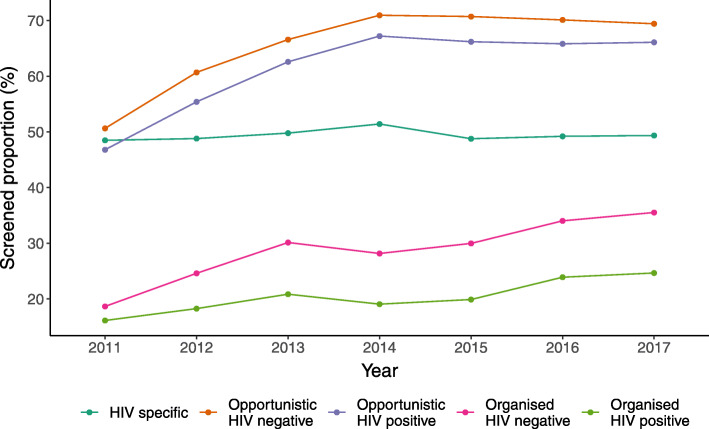
The proportion of women covered annually by the cervical cancer screening programme in Estonia, 2011–2017. WLWH and the general population group were divided into five categories: 1) WLWH, studied according to the HIV screening programme (HIV specific); 2) General population women, studied outside the organised (opportunistic) screening programme (Opportunistic HIV negative); 3) WLWH, studied outside the organised screening programme (Opportunistic HIV positive); 4) General population women, studied according to the general population organised screening programme (Organised HIV negative); and, 5) WLWH, studied according to the general population organised screening programme (Organised HIV positive)

WLWH and general population women aged 30–55 years were considered annually covered by the organised CC screening for 5 years after a health care claim with the ICD-10 code Z12.4 was filed (Fig. 1). This definition follows the principles of organised CC screening in Estonia in terms of target age and screening interval [11].

WLWH and general population women aged 16+ were considered annually covered by the opportunistic CC screening for 5 years starting from the date of a Pap testing episode recorded on a healthcare claim without an ICD-10 code Z12.4 The screening interval mirrors the 5-year period employed in organised screening.”

WLWH were considered to be covered by HIV-specific CC screening for the next 2 years starting from the date when the test was conducted independently of age (16+). There is no special CC screening programme for WLWH in Estonia, but the Estonian Society of Infectious Diseases recommends WLWH should be screened every second year [14].

### Longitudinal coverage

The primary outcome of interest for this study, namely longitudinal coverage with organised CC screening, was defined using a binary variable indicating whether a person has been screened longitudinally over the whole follow up period of the study (yes or no). WLWH and general population women aged 30–55 years were considered longitudinally covered by the organised CC screening if an invoice with the ICD-10 code Z12.4 was filed once every 5 years (with a minimum of two times over the observation period of 9 years).

Second, given the focus of our analysis, we also distinguished specific CC screening needs of WLWH as HIV-specific CC screening.

WLWH (aged 16+ years) were considered longitudinally covered by HIV-specific screening if a Pap test was conducted once every 2 years (at a minimum of two times over the five-year period of observation) (Table 2).

We also analysed factors associated with longitudinal coverage with an organised CC screening coverage among WLWH and general population women; and longitudinal coverage with HIV-specific CC screening among WLWH (Table 2).

#### Other variables (screening predictors)

For all women: age (at the time of cohort inclusion), place of residence - regions of Estonia (capital, north-east and other) were ascertained. For WLWH: HIV stage at index visit based on the ICD-10 code (acute – B23.0; clinical latency - B23.1, Z21; AIDS F02.4, B20-B24; and unknown), AIDS diagnosis at any time of follow-up (defined by the occurrence of, and healthcare claim with, the ICD-10 codes F02.4, or B20-B24), retention in HIV care (defined by at least two HIV-related physician visits within a 12-month period continuously across the whole period of observation [15], comorbidities such as drug abuse (based on ICD-10 diagnoses: F10-F19, T40, Y12), and HCV infection (ICD 10 diagnoses. B17.1, or B18.2).

### Statistical analysis

Descriptive statistics (i.e. proportions, means, standard deviations and medians) for length of follow-up time, number of Pap tests, and population-based annual and longitudinal screening coverages (opportunistic, organised, HIV-specific) are presented. In addition, we characterise the WLWH cohort using HIV stage at index date, AIDS diagnosis at any time of follow-up, drug abuse and infection rates for HCV co-morbidities. The ORs and AORs with 95% CI for predictors of longitudinal coverage were estimated in univariate and multivariate logistic regression analyses (adjusted for region and age at entry to the study and insurance status). All analyses were performed in R (version 3.5.1, 2018).

## Results

### Study population

We identified 2614 women from the EHIF database had received HIV-related health care services between 2009 and 2018. From these WLWH, we excluded 112 who were under 16 years of age, 9 due to previous CC diagnosis, and 47 non-citizens.

In all, 2448 WLWH and 7558 general population women were followed for 17,210 and 70,990 person-years, respectively (Table 1).

**Table 1:** Characteristics of women living with HIV (WLWH) and general population women in Estonia, 2009–2018

Characteristics	WLWH	General population group	***P***-value
N	2448	7558	
Age, mean (SD)	31.2 (11.3)	29.9 (10.9)	
Age groups n,(%)			
16–19	114 (4.7)	612 (8.1)	
20–29	1335 (54.5)	4196 (55.5)	
30–39	563 (23.0)	1587 (21.0)	
40–49	222 (9.1)	647 (8.6)	
50–55	98 (4.0)	252 (3.3)	
56+	116 (4.7)	264 (3.5)	
Follow-up, years mean (SD)	7.03 (3.0)	9.39 (0.8)	
Follow-up time total, person years	17,210.5	70,990.3	
Region (n,%)			
Capital	1009 (41.2)	3104 (41.1)	
North-East	1214 (49.6)	3822 (50.5)	
Other	225 (9.2)	632 (8.4)	
Time to the 1st Pap test after the index date (months) (range, mean, SD; median)	0.1315–120.7; 34.2; 24.8; 25.8	0.0657–119.9; 28.2; 24.5; 21.0	
Uninsured (ever during the follow-up) (%)	744 (30.4)	13 (0.2)	< 0.00001
Drug abuse (%)	360 (14.7)	9 (0.1)	< 0.00001
HCV (%)	916 (37.4)	20 (0.3)	< 0.00001

The mean age of WLWH at the beginning of follow-up was 30.6 (SD 11.8) years and most (over 90%) were from the capital and North-Eastern regions of Estonia. The majority of WLWH (79.8%) were in the ‘clinical latency’ stage at the start of follow-up, and 31% were considered retained in care over the entire follow-up. One-fifth (19.7%) of the WLWH developed AIDS during the study period, 14.7% (*n* = 360) had diagnoses codes indicating drug abuse, and 37.4% (*n* = 916) had concomitant HCV infection.

During the whole follow-up period, 53 women from the general population group were diagnosed with HIV (incidence 0.75 cases per 1000 population).

### Cervical cancer screening

A total of 7304 Pap tests (opportunistic and organised) were linked to 2448 WLWH and 25,078 to 7558 general population women. Overall, 587 (24%) of WLWH and 1269 (16.8%) of general population women had no record of any Pap testing during 2009–2018. 30.4% of WLWH and 0.2% of women in the general population were not insured. 314 (12.8%) WLWH and 914 (12.1%) of general population women (difference in proportions: *p* = 0.336) had only one Pap test conducted during the whole follow-up period. The maximum number of Pap tests per woman during the study period was 20. Mean time between two consecutive Pap tests was 21.0 (median 17.1, SD 13.7, range 0.1–116) and 22.9 (median 19.5, SD 14.6, range 0.1–100.3) months in WLWH and general population women, respectively *p* = 0.00002.

#### Opportunistic screening

The mean annual opportunistic screening coverage was 61.45% among WLWH and 65.59% among the comparator group (*p* < 0.00001) (Fig. 1).

The mean annual opportunistic screening coverage among WLWH meeting the HIV-specific screening target (repeated testing every second year) was 49.4% (Fig. 1). One-quarter (*n* = 595, 24.3%) of WLWH were covered longitudinally by HIV-specific CC screening.

#### Organised screening

The mean annual coverage with organised screening was 20.4 and 28.7% among WLWH and the general population, respectively (*p* < 0.00001) (Fig. 1).

The proportion of women in the general population group and WLWH longitudinally covered by organised screening was 19.01% (95% CI 18.05–19.97) and 13.9% (95% CI 12.35–15.45), respectively (*p* < 0.00001).

### Factors associated with cervical cancer screening longitudinal coverage

Longitudinal coverage with HIV-specific CC screening was inversely associated with age. The oldest WLWH (aged over 56 years) had the lowest odds for coverage AOR 0.136 (95% CI 0.060, 0.281), as had the HCV co-infected AOR 0.754 (95% CI 0.619, 0.916), uninsured AOR 0.331 (95% CI 0.264, 0.412) and those abusing drugs AOR 0.459 (95% CI 0.336, 0.618). WLWH retained in HIV care AOR 1.972 (95% CI 1.615, 2.410) had higher odds of being covered (Table 2).

**Table 2:** Predictors of cervical cancer screening programme period coverage in Estonia, 2009–2018

	WLWH: HIV specific screening (595/2448) 24.31%			WLWH: organised screening (266/1914) 13.90%			General population women: organised screening (1210/6365) 19.01%		
	N (% covered)	OR (95% CI)	AOR (95% CI)	N (% covered)	OR (95% CI)	AOR (95% CI)	N (% covered)	OR (95% CI)	AOR (95% CI)
Region									
Capital	1009 (31.32)	1	1	788 (13.71)	1	1	2618 (20.02)	1	1
North-East	1214 (27.76)	0.704 (0.581,0.851)	**0.705 (0.581,0.855**)	970 (14.85)	0.999 (0.759,1.317)	1.007 (0.764,1.330)	3273 (18.70)	0.920 (0.808,1.048)	0.918 (0.806,1.046)
Else	225 (27.11)	0.662 (0.473,0.915)	0.767 (0.544,1.071)	156 (8.97)	0.560 (0.298,0.978)	0.610 (0.324,1.070)	474 (15.61)	0.739 (0.563,0.959)	**0.732 (0.557,0.951)**
Age groups									
16–19	114 (34.21)	1	1						
20–29	1335 (32.96)	0.937 (0.622,1.424)	0.930 (0.618,1.419)	1031 (16.39)	1	1	3879 (18.72)	1	1
30–39	563 (28.42)	0.691 (0.448,1.078)	0.686 (0.444,1.071)	563 (11.19)	0.617 (0.449,0.838)	0.621 **(0.452,0.843)**	1587 (17.77)	0.939 (0.805,1.091)	0.948 (0.813,1.103)
40–49	222 (23.87)	0.523 (0.315,0.868)	0.521 **(0.314,0.866)**	222 (10.36)	0.554 (0.340,0.865)	0.565 **(0.347,0.883)**	647 (23.65)	1.344 (1.099,1.635)	**1.364 (1.115,1.660)**
50–55	98 (12.25)	0.204 (0.095,0.411)	0.206 **(0.096,0.416)**	98 (11.22)	0.563 (0.278,1.038)	0.586 (0.289,1.083)	252 (19.44)	1.047 (0.751,1.433)	1.083 (0.776,1.484)
56+	116 (8.62)	0.136 (0.060,0.281)	0.135 **(0.060,0.281)**						
Insured (uninsured if it occurs ever during the follow-up) (baseline is insured)	1704 (34.04) 744 (18.01)	1 0.426 (0.343,0.525)	1 **0.331 (0.264,0.412)**	1339 (15.53) 575 (10.09)	1 0.610 (0.444,0.826)	1 **0.545 (0.394,0.745)**	6355 (19.02) 10 (10.00)	1 0.4729 (0.026,2.520)	1 0.477 (0.026,2.545)
Stage at index visit (n %)									
Clinical latency	1955 (29.67)	1	1	1548 (13.44)	1	1			
Acute	87 (39.08)	1.340 (0.851,2.085)	1.444 (0.908,2.271)	69 (23.19)	1.794 (0.974,3.136)	**1.993 (1.073,3.523)**			
AIDS	382 (24.35)	0.689 (0.531,0.887)	0.874 (0.666,1.138)	278 (14.03)	1.005 (0.686,1.441)	1.198 (0.811,1.734)			
Unknown	24 (29.17)	0.885 (0.338,2.085)	1.069 (0.399,2.602)	19 (15.79)	1.178 (0.272,3.586)	1.331 (0.304,4.131)			
AIDS dgn at any time of follow-up (baseline is AIDS not diagnosed)	1383 (31.67) 483 (27.54)	1 0.874 (0.690,1.102)	1 0.879 (0.691,1.114)	1066 (14.92) 400 (10.75)	1 0.708 (0.489,1.006)	1 **0.678 (0.466,0.966)**			
HIV care retention (yes: n %) (baseline is not retained)	1691 (25.43) 757 (37.52)	1 1.751 (1.453,2.108)	1 **1.972 (1.615,2.410)**	1324 (14.50) 590 (12.54)	1 0.824 (0.614,1.095)	1 0.939 (0.688,1.273)			
Drug abuse (yes: n, %) (baseline is no drug abuse)	2088 (31.37) 360 (16.39)	1 0.517 (0.380,0.693)	1 **0.459 (0.336,0.618)**	1624 (14.84) 290 (8.62)	1 0.600 (0.379,0.914)	1 **0.543 (0.342,0.830)**	6360 (19.03) 5 (0.00)	1 0 (−)	1 0 (−)
HCV infection (yes: n %) baseline is no HCV	1532 (31.27) 916 (25.66)	1 0.868 (0.718,1.048)	1 **0.754 (0.619,0.916)**	1164 (14.26) 750 (13.33)	1 0.998 (0.759,1.307)	1 0.844 (0.637,1.115)	6355 (18.98) 10 (40.00)	1 3.450 (0.845,13.217)	1 3.500 (0.856,13.418)
Drug abuse or HCV co-infection	1448 (32.46) 1000 (24.40)	1 0.780 (0.646,0.940)	1 **0.683 (0.562,0.828)**	1092 (15.11) 822 (12.28)	1 0.856 (0.651,1.122)	1 0.732 **(0.552,0.965)**	6353 (18.98) 12 (33.33)	1 2.703 (0.682,9.567)	1 2.751 (0.693,9.751)

In WLWH, organised CC screening programme uptake was inversely associated with age 30–39 years old AOR 0.621 (95% CI 0.452, 0.843), 40–49 years old AOR 0.565 (95% CI 0.347, 0.883), drug abuse AOR 0.543 (95% CI 0.342, 0.830) and not having insurance AOR 0.545 (95% CI 0.394, 0.745). Those in the ‘acute HIV’ stage were more likely to be screened AOR 1.993 (95% CI 1.073, 3.523).

In general population women, longitudinal coverage with organised screening was inversely associated with living in a region other than the capital and the north-east AOR 0.732 (95% CI 0.557, 0.951). 40–49 year olds were more likely to be covered AOR 1.364 (95% CI 1.115, 1.660).

## Discussion

This study provides CC coverage data and comparison among HIV-infected women and the general population in Estonia.

To our knowledge, this is the first population-based study assessing CC screening coverage among WLWH in an East European country with a characteristic HIV epidemic. Our results highlight unacceptably low screening coverage of WLWH.

The annual screening coverage found in the present study is lower than that found in previous studies of HIV (France 76.5% [16] and Italy 61% [17]). These findings suggest that a substantial proportion of HIV infected women in Estonia are under-screened, which is in line with the findings from a Danish study [18]. However, the difference might also reflect the methodologies used to measure coverage. Studies based on self-reported data are more likely to overestimate screening.

There is an increasing need of evidence as to whether targeted preventive and treatment guidelines are necessary for the management of HIV-infected patients. We choose to include data on the general population of women in this analysis so as to determine barriers that WLWH face in excess of what would be expected. This data is essential to minimise vulnerability to HIV, to eliminate inequities in the HIV CC care cascade, to reduce vulnerabilities to poor outcomes, and to improve health and well-being. We found that almost a quarter (24.0%) of WLWH in contact with healthcare services had no Pap testing undertaken in comparison to less than one-fifth in the general population of women. For the whole study period, a quarter of WLWH (24.3%) were covered with the opportunistic Pap testing that met the HIV-specific CC screening target. Longitudinal coverage of WLWH with organised screening was two times lower (13.9%). While exceedingly few women in the general population were uninsured, one-third (30.4%) of WLWH lacked health insurance over the study period. It is of importance to note that while HIV care in Estonia is free for those in need, this does not extend to all healthcare and prevention services. For example, only women covered with health insurance are invited to organised CC screening. CC screening coverage among WLWH in Estonia is unacceptably low and significantly lower than that in the general population in our study.

Unlike the general population of women, coverage with organised screening tended to decrease with age among WLWH, and was highly affected by insurance status and concomitant drug use (or HCV infection) with one exception: WLWH co-infected with HCV had significantly lower odds of being covered than those without HCV.

In the present study, younger age was a predictor for screening attendance by WLWH, which is in line with a study from Canada by Burchell, et al. [19]. This may reflect differences in health behaviour, and awareness of the importance of screening programmes. WLWH retained in HIV care were more likely to be screened, a finding also documented in the UK [20]. This may be explained by health beliefs and the behaviour of women, and by the opportunity to engage women in HIV care into preventive services. Our findings highlight barriers to CC screening among WLWH. Consistent with previous studies, we found that drug abuse [21] and a lack of access (through not having insurance) [22] are the main barriers. In line with Elfström, et al. [23], we found that organised screening uptake by the general population of women in Estonia was low (< 20%), in parallel with the relatively high (65%) uptake of opportunistic screening. Our findings support the worrisome fact that women are more likely to be Pap tested opportunistically, which has been shown to provide no additional benefit to preventing cancer [24]. While the screening coverage rates are importantly lower in the WLWH population, the overall screening rate in Estonia is very low (< 30% in both the general population and the WLWH population), and should prompt urgent action. Screening program organization could be improved by personal invitation and developing tailored messages (also in terms of the access: sms messages, emails, mailed letters), recall system of invitation. Several barriers as the perceptions of women with regard to the benefits of prevention, of low efficacy of cancer screening, the anxiety about the results should be addressed though awareness raising campaigns and initiatives.

Given our findings, there is a clear need to improve CC screening attendance among women in Estonia. We did not assess the performance of the Pap test in our study, but there are indirect indications of significant under-detection/diagnosis of CIN2+ in Estonia [25]. However, we support the idea of seeking and testing new innovative interventions to improve programme uptake through interventions such as self-sampling and electronic reminders [26], as well as changing CC test screening to HPV DNA.

The strength of our analysis lies in the use of population-based data free of individual recall / social desirability bias. We had a sufficient sample size to derive credible and precise estimates over the long follow-up period. With this data, we were able to assess continuous coverage with screening that allowed us to evaluate the screening behaviour longitudinally in WLWH and in the general population for 10 years. It is important to have a longitudinal life-course perspective in CC screening.

Our study had some limitations. Using administrative health insurance data has drawbacks. While HIV care is free for all in need, CC screening is only provided for those who are insured. This could potentially lead to an overestimation of all coverage rates in the study. But it is likely that this does not significantly affect the interpretation of our results given that being uninsured had the strongest effect on CC screening non-adherence – both among WLWH and among the general population. Ignoring the impact of pregnancy, dysplasia and CIN treatment in the coverage estimation, our results are prone to overestimation of coverage. Furthermore, there might be some misclassification of the use of the ICD diagnosis code for organised CC screening. However, we are not aware of any data helping to quantify this effect. We were not able to control for potential (known) confounders (income, education, occupation, ART treatment) for both the coverage estimates and measures of coverage association, as this data is not available in the EHIF database. Given that we have only data beginning from the year 2009, it is likely that some subjects who underwent a Pap testing in years 2007–2008 might have been misclassified, resulting in a slight underestimation of coverage in the early study years. In addition, 30.4% of WLWH didn’t have health insurance and could introduce a potential bias. Although we believe this fact doesn’t invalidate our findings, because uninsured general population women were less covered by screening program as well. “In addition, we saw a significant difference in health insurance coverage among general population women and WLWH. Yet, we do not believe this fact invalidate our findings, as uninsured general population women were also less covered by screening program.

However, such potential limitations seem unlikely to account for the clear patterns observed in this study, and we believe that our study provides informative results that allow inferences to be made for other populations of European WLWH.

## Conclusion

In conclusion, our findings provide new evidence to address barriers to CC screening in WLWH. The CC screening coverage among WLWH in Estonia is unacceptably low. Being uninsured has the strongest effect, both among WLWH and in the general population. This barrier needs urgent attention. Given the efficacy of ART, the increased risk of CC among WLWH, and the low coverage of screening in several countries with high HIV prevalence, integrating CC screening with HIV healthcare services is needed. WLWH with concomitant comorbidities need further attention to assure state of the art health care and cancer prevention.

## Data Availability

The data that support the findings of this study are available from Estonian Health Insurance Fund but restrictions apply to the availability of these data, which were used under license for the current study, and so are not publicly available. Data are however available from the corresponding author upon reasonable request and with permission of Estonian Health Insurance Fund.

## Abbreviations

AIDS: Acquired immunodeficiency syndrome
AOR: Adjusted odds ratio
ART: Antiretroviral therapy
CC: Cervical cancer
CIN: Cervical intraepithelial neoplasia
EHIF: Estonian Health Insurance Fund
HCV: Hepatitis C virus
HIV: Human immunodeficiency virus
HPV: Human papillomavirus
ICD-10: 10th revision of the International Statistical Classification of Diseases and Related Health Problems
OR: Odds ratio
WLWH: Women living with HIV
